# Characterization of baseline hemodynamics after the Fontan procedure: a retrospective cohort study on the comparison of 4D Flow MRI and computational fluid dynamics

**DOI:** 10.3389/fphys.2023.1199771

**Published:** 2023-05-25

**Authors:** Gyu-Han Lee, Hyun Jung Koo, Kyung Jin Park, Dong Hyun Yang, Hojin Ha

**Affiliations:** ^1^ Department of Interdisciplinary Program in Biohealth-Machinery Convergence Engineering, Kangwon National University, Chuncheon, Republic of Korea; ^2^ Department of Radiology, Asan Medical Center, Research Institute of Radiology, University of Ulsan College of Medicine, Seoul, Republic of Korea; ^3^ Department of Electrical and Electronic Engineering, Yonsei University, Seoul, Republic of Korea; ^4^ Department of Smart Health Science and Technology, Kangwon National University, Chuncheon, Republic of Korea

**Keywords:** Fontan circulation, 4D Flow MRI, computational fluid dynamics, blood flow distribution, hemodynamics

## Abstract

**Introduction:** The aim of this study was to characterize the hemodynamics of Fontan patients using both four-dimensional flow magnetic resonance imaging (4D Flow MRI) and computational fluid dynamics (CFD).

**Methods:** Twenty-nine patients (3.5 ± 0.5 years) who had undergone the Fontan procedure were enrolled, and the superior vena cava (SVC), left pulmonary artery (LPA), right pulmonary artery (RPA), and conduit were segmented based on 4D Flow MRI images. Velocity fields from 4D Flow MRI were used as boundary conditions for CFD simulations. Hemodynamic parameters such as peak velocity (Vmax), pulmonary flow distribution (PFD), kinetic energy (KE), and viscous dissipation (VD) were estimated and compared between the two modalities.

**Results and discussion:** The Vmax, KE, VD, PFD_Total to LPA_, and PFD_Total to RPA_ of the Fontan circulation were 0.61 ± 0.18 m/s, 0.15 ± 0.04 mJ, 0.14 ± 0.04 mW, 41.3 ± 15.7%, and 58.7 ± 15.7% from 4D Flow MRI; and 0.42 ± 0.20 m/s, 0.12 ± 0.05 mJ, 0.59 ± 0.30 mW, 40.2 ± 16.4%, and 59.8 ± 16.4% from CFD, respectively. The overall velocity field, KE, and PFD from the SVC were in agreement between modalities. However, PFD from the conduit and VD showed a large discrepancy between 4D Flow MRI and CFD, most likely due to spatial resolution and data noise. This study highlights the necessity for careful consideration when analyzing hemodynamic data from different modalities in Fontan patients.

## 1 Introduction

The Fontan procedure is a surgical treatment for patients with single ventricle physiology, which involves redirecting venous blood directly to the pulmonary circulation, bypassing the right ventricle. In the Fontan circulation, it is crucial to have efficient blood flow distribution and minimal energy loss to prevent complications (Rijnberg et al., 2018).

4D Flow MRI enables the quantification of hemodynamic parameters such as kinetic energy (KE), pulmonary flow distribution (PFD), and energy loss which can aid in understanding the hemodynamics of Fontan circulation (van der Woude et al., 2021; Rijnberg et al., 2022a; Weiss et al., 2023). Furthermore, 4D Flow MRI has been used to identify the correlations between hemodynamic energetics and clinical outcomes in Fontan circulation (Kamphuis et al., 2019; Kamphuis et al., 2021; Rijnberg et al., 2022b). However, the accuracy of 4D Flow MRI data is limited by low spatiotemporal resolution and signal-to-noise ratio issues, which can impact the reliability of the results.

To compensate for this, some studies have used CFD in conjunction with 4D Flow MRI, which has shown a correlation between hemodynamic energy loss in Fontan circulation and various factors, such as exercise intolerance, lower systemic flow, and cardiac index (Haggerty et al., 2014; Khiabani et al., 2014). Efficient blood flow distribution is especially critical for infants immediately after undergoing the Fontan procedure. However, most studies have been conducted on a wide age range of patients, from childhood to adulthood (Haggerty et al., 2014; Khiabani et al., 2014; McLennan et al., 2019; Rijnberg et al., 2021; Frieberg et al., 2022; Weiss et al., 2023), and there is a lack of baseline characterizations of infants and their correlation to follow-up results.

In this study, we present baseline hemodynamics of patients immediately following the Fontan procedure and investigate the correlations and discrepancies among hemodynamics parameters obtained from 4D Flow MRI and CFD.

## 2 Materials and methods

### 2.1 Study population

The study was approved by the Institutional Review Board and the approval number is IRB 2018-0404, and involved a total of 29 consecutive patients who received the Fontan procedure and underwent cardiac magnetic resonance (CMR) between February 2017 and December 2019. The goal of CMR was assessment of cardiac function and patency of the Fontan conduit following surgical treatment. As part of routine CMR protocol, 4D Flow MRI was also performed. All Fontan patients included in this study underwent extracardiac conduit surgery using an 18 mm Gore-Tex Stretch vascular graft to redirect venous blood flow from the inferior vena cava to the pulmonary arteries. Fenestration was performed in three out of the total number of patients. Fontan operation is completed for 3- or 4-year-olds with a consistent protocol. During the Fontan procedure, all patients underwent sedation without the need for intubation or mechanical ventilation under the supervision of an anesthesiology specialist, and sedation was generally achieved by intravenously administering propofol (1 mg/kg). Patient demographic information is provided in Table 1.

**TABLE 1:** Patient demographic details.

Characteristics	Information
Age (year)	3.5 ± 0.5
Interval between Fontan operation and 4D Flow MRI (months)	5.1 ± 5.9
Body surface area (m^2^)	0.6 ± 0.0
Gender (male/female)	17/12
Connection type (intra-atrial/extracardiac/atriopulmonary)	0/29/0
Bilateral superior vena cava connections	2
Stroke volume of functional single ventricle, ml	23.8 ± 6.3
Cardiac index (L/min/m^2^)	3.1 ± 0.5

### 2.2 4D Flow MRI

Clinical 1.5T and 3.0T MRI scanners (Philips Ingenia; Philips Healthcare, Best, Netherlands) were used to acquire 4D Flow MRI scans, employing a retrospectively cardiac-gated gradient-echo sequence with four-point asymmetric flow encoding. The 4D Flow MRI data were acquired following injection of a Gd contrast agent (Magnevist; Bayer Healthcare Pharmaceuticals, Berlin, Germany). Scans were performed during free breathing. Scan parameters included: VENC = 40–150 cm/s (40–80 cm/s used in 28 cases, and 150 cm/s used in 1 case), flip angle 15°, echo time = 2.5–4.2 ms, repetition time = 3.9–6.8 ms. The acquired temporal resolution was 20–42 ms. The 3D field of view = 510–576 × 512–576 × 40–56 mm^3^ and matrix size = 256–288 × 256–288 × 20–28 were adjusted depending on each subject’s anatomy to ensure coverage of the entire SVC, LPA, RPA, and conduit, while maintaining an acquired voxel size of 2.0 × 2.0 × 2.0 mm^3^. Total scan time was approximately 10 min.

### 2.3 Computational fluid dynamics

Fontan circulation was simulated using Fluent (v.2021 R1; ANSYS, Inc., Canonsburg, United States). The fluid was assumed Newtonian fluid, modelled with a density and dynamic viscosity of 1060 kg/m^3^ and 0.0035 kg/m s (3.5 cP), respectively. In 4D Flow MRI data, the planes of each region adjacent to the center of the geometry were cut, from which time-averaged flow rate data were obtained. The time-averaged flow rate was applied as a mass flow inlet for the SVC and a mass flow outlet for the LPA and RPA, and the conduit was applied as a pressure-inlet as atmospheric pressure for mass conservation. The rigid body assumption and no-slip condition were used for the vessel wall. A dependency test was performed by changing the element size from 0.7 to 0.3 mm for any three cases, and the simulation was performed by selecting 0.4 mm, the size in which the increase rate of total VD was less than 5% for all cases (Supplementary Figure S1). All meshes were made of polyhedral mesh. The simulation was performed using a steady-state condition, and incompressible Reynolds-averaged Navier–Stokes equations were solved using the coupled scheme to resolve pressure-velocity coupling. The shear stress transport k-omega turbulence model was used, and a second-order upwind scheme was used for momentum and turbulence equations. The all-residual criteria were set to 1e-6.

### 2.4 Pre- and post-processing

**FIGURE 1:**
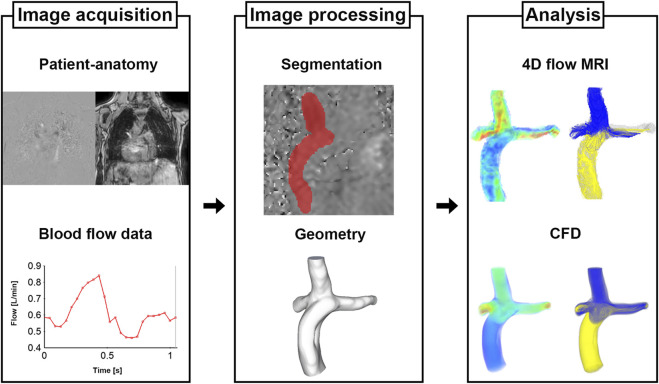
Process of cardiovascular analysis for Fontan patients.

The process for Fontan analysis is shown in Figure 1. The 4D Flow MRI images were converted from the DICOM to Nifti format via MATLAB (v.R2020a; The MathWorks, Inc., Natick, United States). The magnitude and velocity images were imported into ITK-SNAP (v.3.8.0; www.itksnap.org) and manual segmentations were performed to extract the patient-specific geometries of total cavopulmonary connection anastomosis. The segmented surface geometry was smoothed using Meshmixer (v.3.5, Autodesk, Inc., San Rafael, United States) to the extent that the diameters remained unchanged. Ensight (v.2021 R1; ANSYS, Inc., Canonsburg, United States) and MATLAB were used to quantify and visualize the hemodynamic parameters.

In this study, we used the following equations to quantify the hemodynamic parameters.

1. Kinetic energy (KE, per unit volume) (Sjöberg et al., 2017; Weiss et al., 2023):

$$\emptyset_{KE}=\frac{1}{2}\rho \left({u_{x}}^{2}+{u_{y}}^{2}+{u_{z}}^{2}\right)\;\left[J/m^{3}\right]$$ (1)

$$\emptyset_{KE\_Total}=\int_{N_{Cell}}\emptyset_{KE}dV\;\left[J\right]$$ (2)

Where ρ denotes the density, u is the time-averaged velocity component of the fluid (either x, y, or z directions), $\emptyset_{KE}$ is the kinetic energy per voxel volume or cell volume, $N_{Cell}$ is the number of voxels or cells within the field of view.

2. VD (per unit volume) (Cibis et al., 2015; Wei et al., 2017; Weiss et al., 2023):

$$\emptyset_{VD}=\mu [2{\left(\frac{\partial u_{x}}{\partial x}\right)}^{2}+2{\left(\frac{\partial u_{y}}{\partial y}\right)}^{2}+2{\left(\frac{\partial u_{z}}{\partial z}\right)}^{2}+{\left(\frac{\partial u_{y}}{\partial x}+\frac{\partial u_{x}}{\partial y}\right)}^{2}+{\left(\frac{\partial u_{z}}{\partial y}+\frac{\partial u_{y}}{\partial z}\right)}^{2}{+\left(\frac{\partial u_{x}}{\partial z}+\frac{\partial u_{z}}{\partial x}\right)}^{2}]\;\left[W/m^{3}\right]$$ (3)

$$\emptyset_{VD\_Total}=\int_{N_{Cell}}\emptyset_{VD}dV\;\left[W\right]$$ (4)

Where μ denotes the dynamic viscosity, u is the time-averaged velocity component of the fluid (either x, y, or z directions), $\emptyset_{VD}$ is the viscous dissipation per voxel volume or cell volume, $N_{Cell}$ is the number of voxels or cells within the field of view.

**FIGURE 2:**
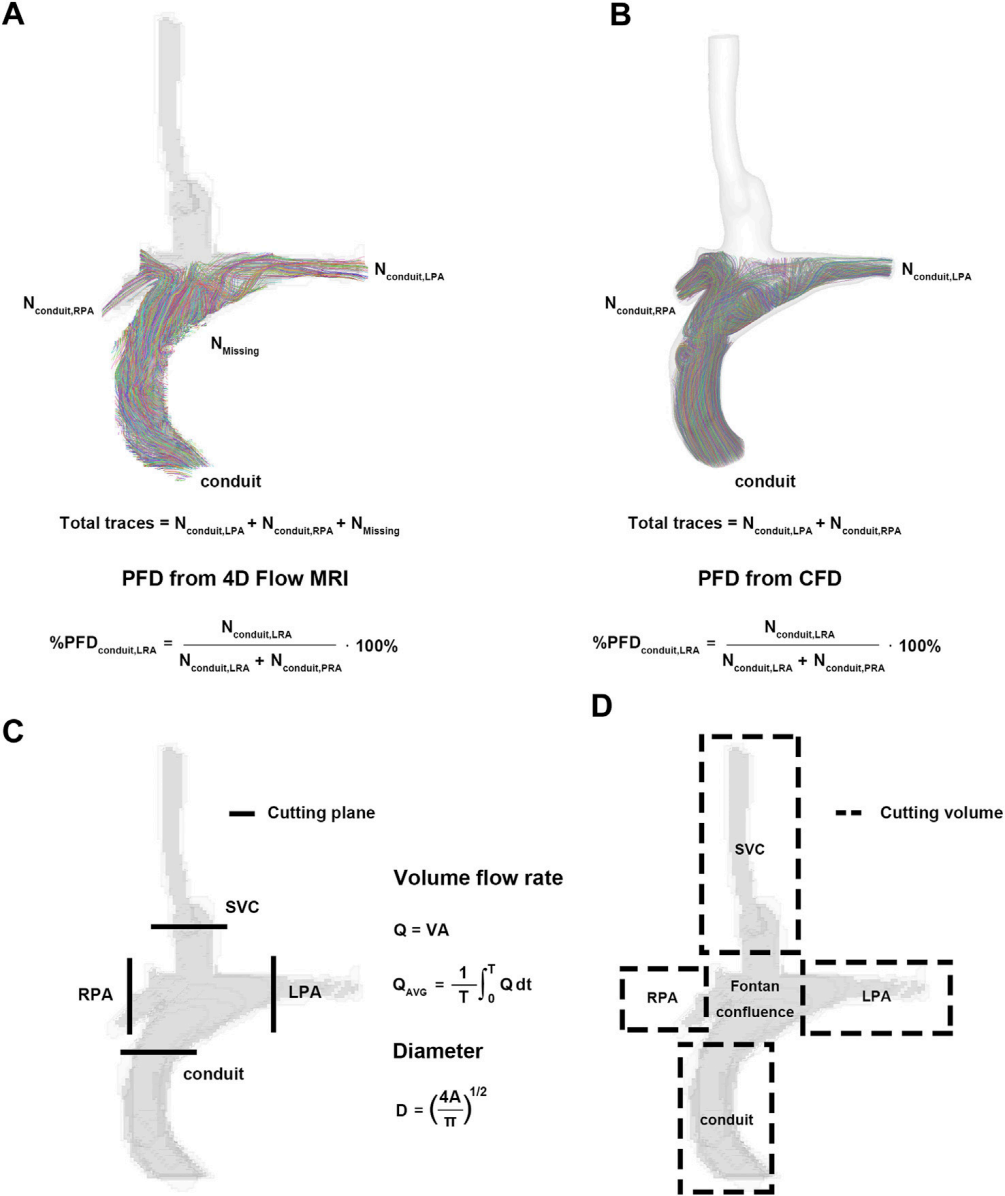
Pre- and post-processing for parameters. **(A)** Pulmonary flow distribution of 4D Flow MRI. **(B)** Pulmonary flow distribution of CFD. **(C)** Cutting plane for flow rate and diameter. **(D)** Cutting volume for mean velocity, kinetic energy, and viscous dissipation. CFD, computational fluid dynamics.

3. Pulmonary flow distribution (PFD) from the SVC or conduit to the LPA or RPA (Figures 2A, B) (Bächler et al., 2013; Ha et al., 2019; Weiss et al., 2023):

$${PFD}_{conduit,LPA}=\frac{N_{conduit,LPA}}{N_{conduit,LPA}+N_{conduit,RPA}}∙100\;\left[\%\right]$$ (5)

Where $N_{A,B}$ is the number of traces from A to B.

### 2.5 Statistical analysis

Statistical analyses were performed using MATLAB. To analyze correlations between 4D Flow MRI and CFD results, a linear regression was used. The Bland-Altman method was then used to calculate accuracy and precision as mean ± standard deviation (SD) of the measured difference against the mean of the results, and to plot the difference between the VD and KE measured by both observers for intra- and inter-observer reliability (Supplementary Figures S2, S3). All differences with *p* < 0.05 were considered significant.

## 3 Results

### 3.1 Kinetic energy and viscous dissipation

**FIGURE 3:**
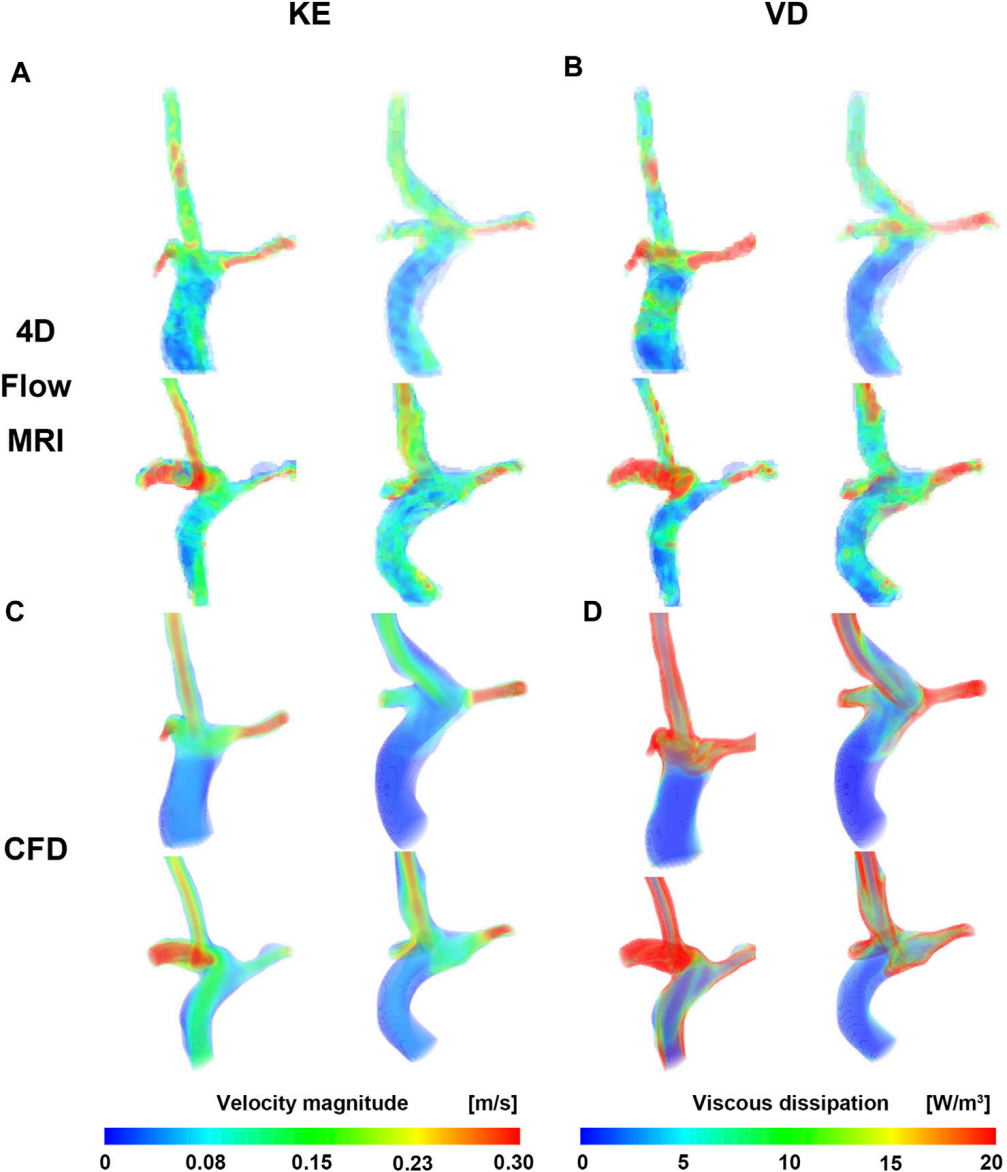
Velocity and viscous dissipation contour. **(A)** Velocity of CFD. **(B)** Viscous dissipation of CFD. **(C)** Velocity of 4D Flow MRI. **(D)** Viscous dissipation of 4D Flow MRI. CFD, computational fluid dynamics.

**FIGURE 4:**
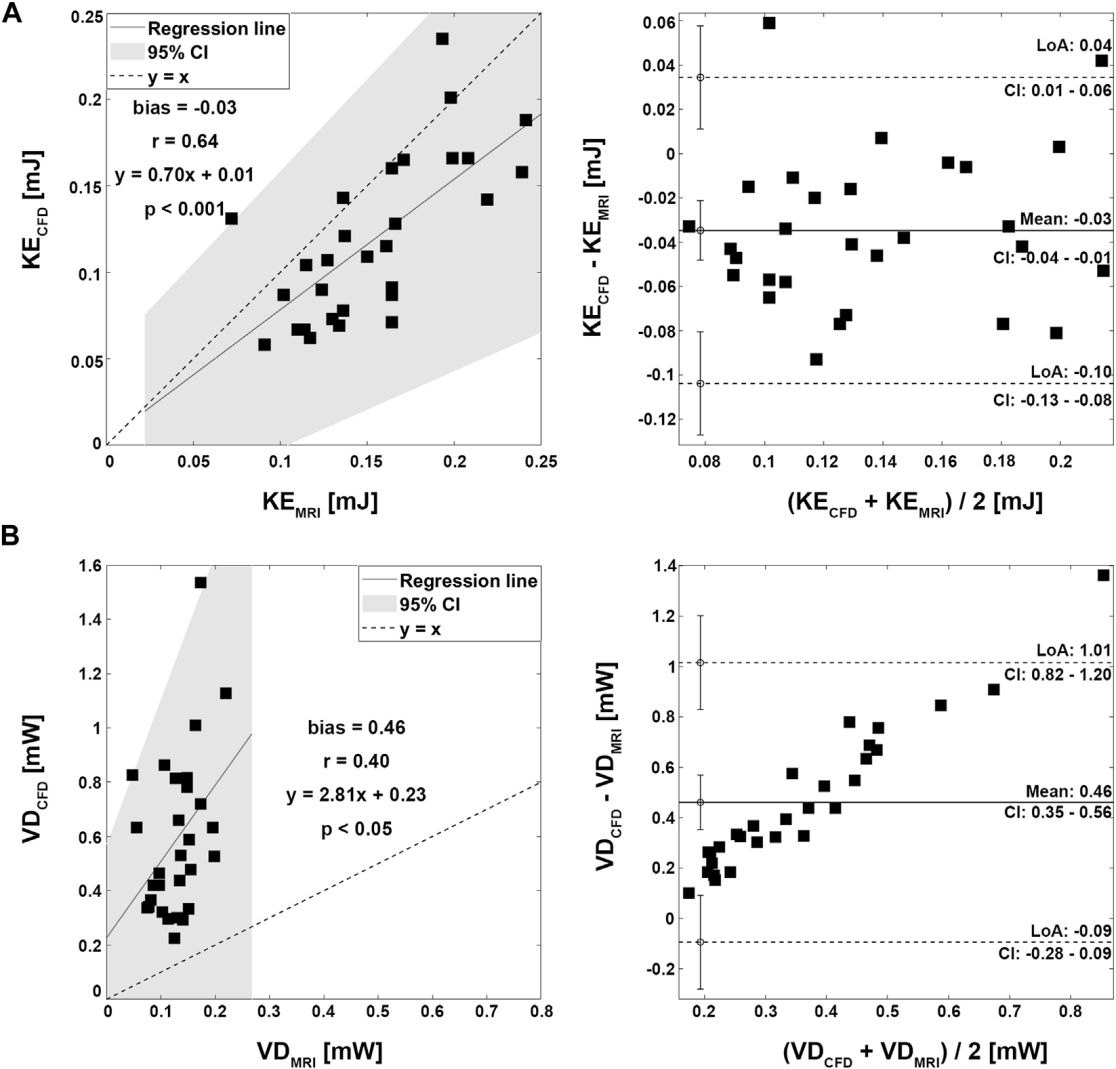
Correlation and Bland-Altman plot for kinetic energy and viscous dissipation. **(A)** Kinetic energy. **(B)** Viscous dissipation.

Velocity fields, V_max_, and KE from 4D Flow MRI and CFD showed qualitatively similar tendencies (Figures 3A, C; Table 2). The linear regression analysis of KE between 4D Flow MRI and CFD showed r = 0.64, y = 0.70x+0.01, bias = −0.03, and *p* < 0.001, respectively. The Bland-Altman limits of agreement of KE from the 4D Flow MRI compared to that from CFD was −0.03 ± 0.07 mJ (Figure 4A). The mean ± SD of V_max_ of 4D Flow MRI and CFD were 0.61 ± 0.18 m/s and 0.42 ± 0.20 m/s, respectively, and those for KE were 0.15 ± 0.04 and 0.12 ± 0.05 mJ, respectively (Table 2).

**TABLE 2:** Results of hemodynamic parameters for 4D Flow MRI and CFD.

Case	4D Flow MRI			CFD		
	V_max_ [m/s]	KE [mJ]	VD [mW]	V_max_ [m/s]	KE [mJ]	VD [mW]
Case 1	0.49	0.16	0.12	0.51	0.16	0.86
Case 2	0.43	0.20	0.13	0.38	0.20	0.81
Case 3	0.30	0.13	0.09	0.27	0.11	0.37
Case 4	0.45	0.17	0.15	0.48	0.17	0.82
Case 5	0.35	0.10	0.08	0.24	0.09	0.34
Case 6	0.48	0.12	0.10	0.31	0.09	0.46
Case 7	0.71	0.16	0.16	0.35	0.09	0.33
Case 8	0.46	0.20	0.16	0.45	0.17	0.78
Case 9	0.58	0.16	0.15	0.30	0.07	0.29
Case 10	0.58	0.13	0.14	0.31	0.07	0.30
Case 11	0.63	0.11	0.13	0.26	0.07	0.22
Case 12	0.52	0.14	0.11	0.33	0.08	0.32
Case 13	0.49	0.24	0.21	0.32	0.16	0.53
Case 14	1.01	0.14	0.17	1.23	0.12	1.01
Case 15	0.55	0.09	0.10	0.43	0.06	0.42
Case 16	0.82	0.21	0.18	0.42	0.17	0.72
Case 17	0.46	0.14	0.06	0.47	0.14	0.63
Case 18	0.82	0.17	0.16	0.34	0.13	0.59
Case 19	0.36	0.07	0.05	0.42	0.13	0.83
Case 20	0.73	0.13	0.15	0.36	0.07	0.44
Case 21	0.56	0.12	0.09	0.26	0.10	0.42
Case 22	0.90	0.24	0.23	0.48	0.19	1.13
Case 23	0.62	0.16	0.16	0.31	0.09	0.48
Case 24	0.73	0.16	0.14	0.44	0.11	0.53
Case 25	0.80	0.12	0.12	0.32	0.06	0.30
Case 26	0.66	0.11	0.08	0.40	0.07	0.34
Case 27	0.67	0.15	0.14	0.40	0.11	0.66
Case 28	0.76	0.22	0.20	0.43	0.14	0.63
Case 29	0.86	0.19	0.19	0.87	0.24	1.54
Mean ± SD	0.61 ± 0.18	0.15 ± 0.04	0.14 ± 0.04	0.42 ± 0.20	0.12 ± 0.05	0.59 ± 0.30

CFD, computational fluid dynamics; KE, kinetic energy; V_max_, peak velocity; VD, viscous dissipation.

The VD from CFD were higher for all cases compared to those from 4D Flow MRI (Figures 3B, D; Table 2). The linear regression analysis of VD between 4D Flow MRI and CFD showed r = 0.40, y = 2.81x + 0.23, bias = 0.46, and *p* < 0.05, respectively. The Bland-Altman limits of agreement of VD from the 4D Flow MRI compared to CFD was −0.46 ± 0.55 mW (Figure 4B). The mean ± SD of VD of 4D Flow MRI and CFD were 0.14 ± 0.04 and 0.59 ± 0.30 mW, respectively (Table 2).

### 3.2 Pulmonary flow distribution

**FIGURE 5:**
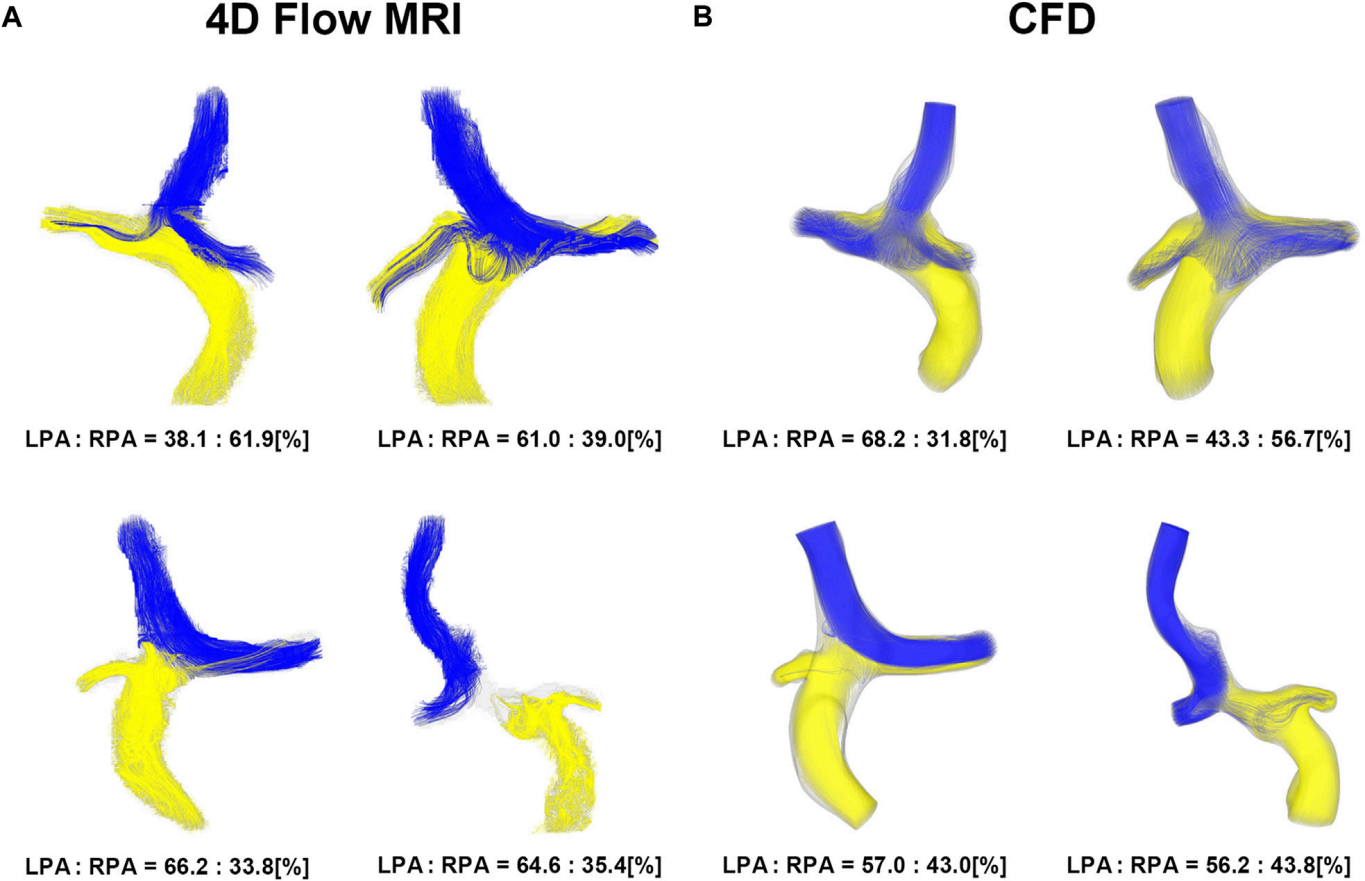
Visualization of pulmonary flow distribution. **(A)** 4D Flow MRI. **(B)** CFD. CFD, computational fluid dynamics.

**FIGURE 6:**
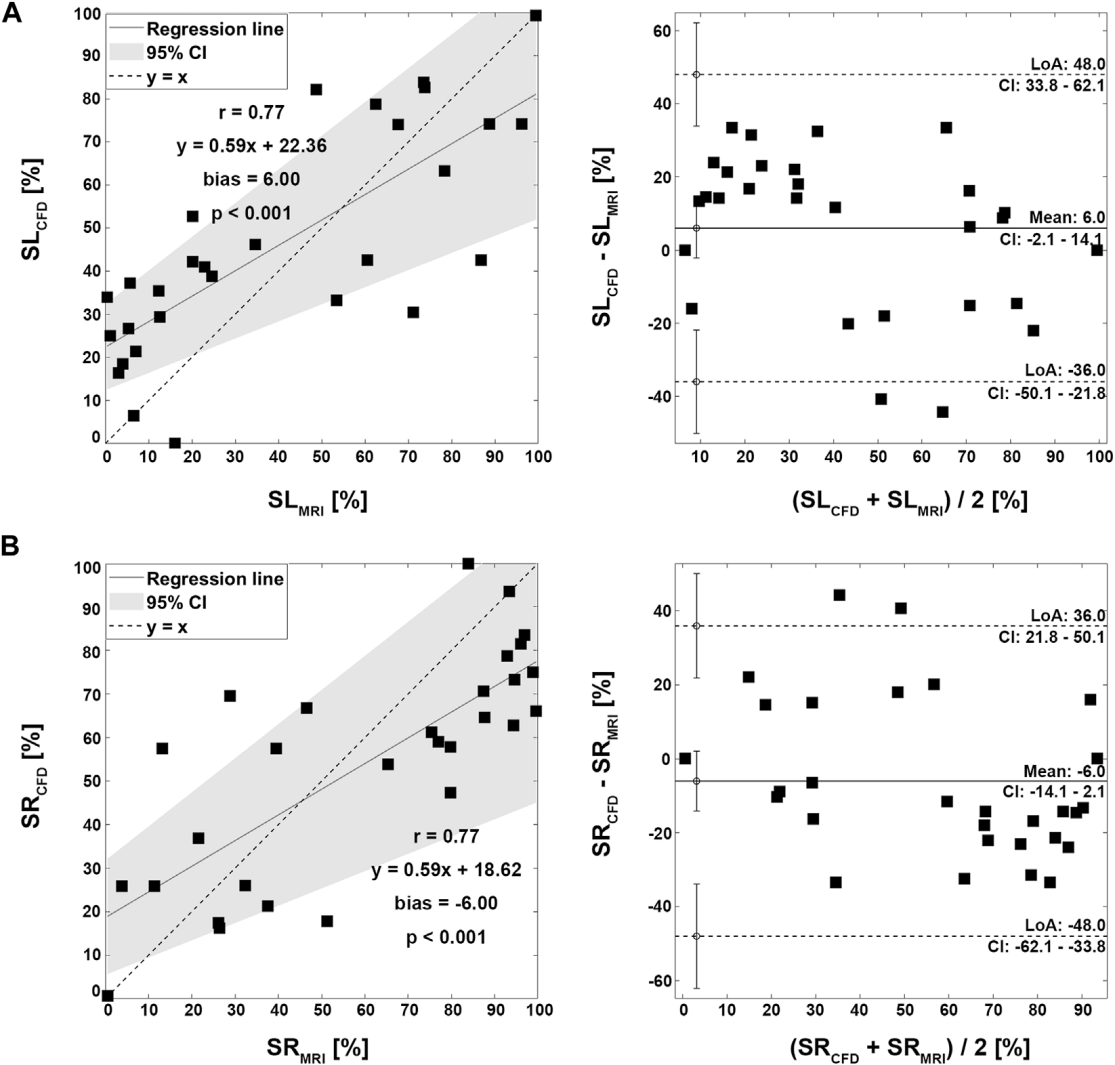
Correlation and Bland-Altman plot for pulmonary flow distribution. **(A)** SL; SVC to LPA. **(B)** SR; SVC to RPA. SVC; superior vena cava, LPA; left pulmonary artery, RPA; right pulmonary artery.

**FIGURE 7:**
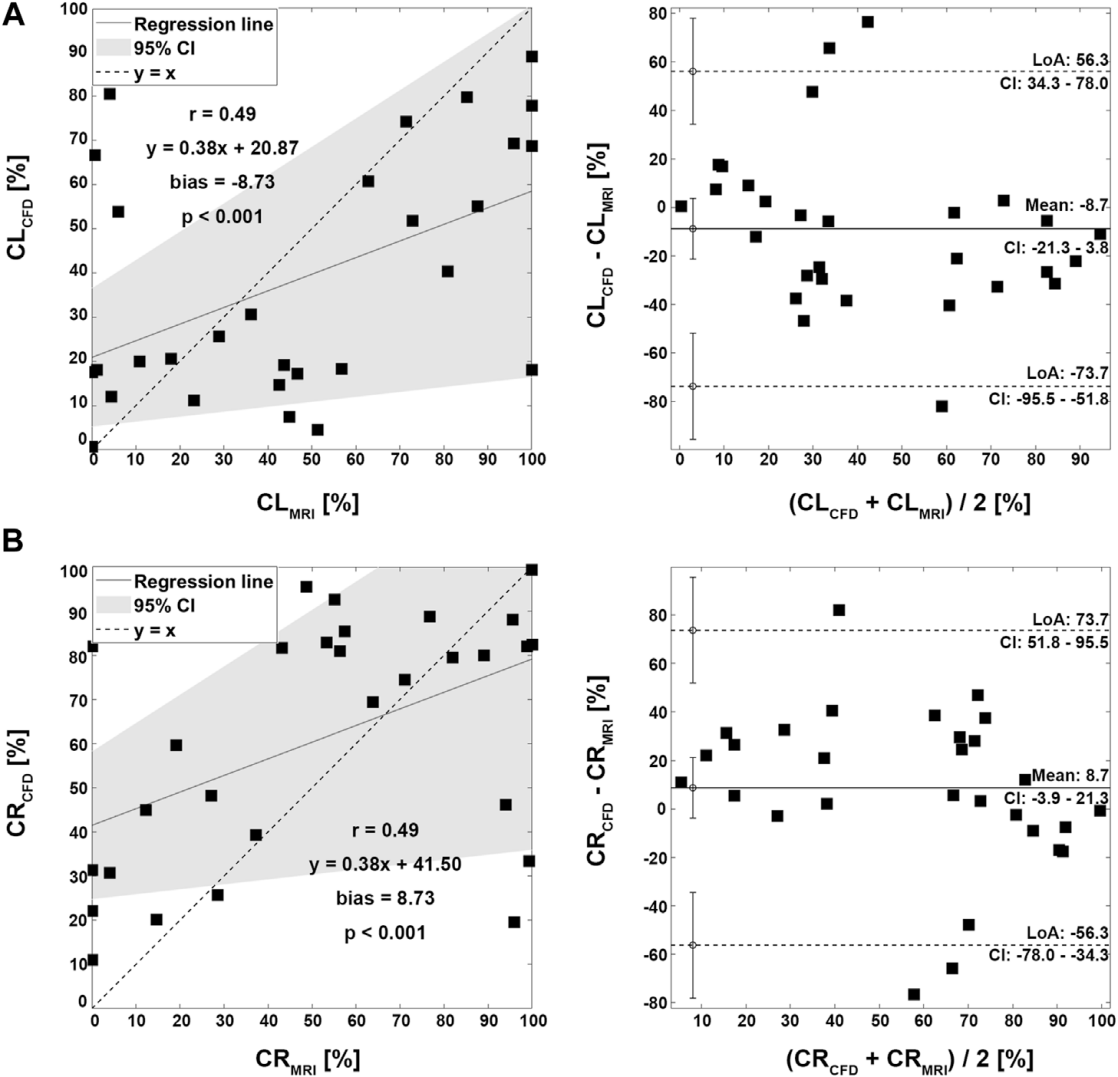
Correlation and Bland-Altman plot for pulmonary flow distribution. **(A)** CL; conduit to LPA. **(B)** CR; conduit to RPA. LPA; left pulmonary artery, RPA; right pulmonary artery.

Four representative cases of PFD visualization for Fontan circulation are shown in Figure 5, and PFD data for all cases are summarized in Table 3. The linear regression analysis of PFD_SVC to LPA_ between 4D Flow MRI and CFD showed r = 0.77, y = 0.59x + 22.36, bias = 6.00, and *p* < 0.001, respectively. The Bland-Altman limits of agreement of PFD_SVC to LPA_ from the 4D Flow MRI compared to CFD was 6.0% ± 42.0% (Figure 6A). The linear regression analysis of PFD_SVC to RPA_ between 4D Flow MRI and CFD showed r = 0.77, y = 0.59x + 18.62, bias = −6.00, and *p* < 0.001, respectively. The Bland-Altman limits of agreement of PFD_SVC to RPA_ from the 4D Flow MRI compared to CFD was −6.0% ± 42.0% (Figure 6B). The linear regression analysis of PFD_conduit to LPA_ between 4D Flow MRI and CFD showed r = 0.49, y = 0.38x + 20.87, bias = −8.73, and *p* < 0.001, respectively. The Bland-Altman limits of agreement for PFD_conduit to LPA_ from the 4D Flow MRI compared to CFD was −8.7% ± 42.0% (Figure 7A); the linear regression analysis for PFD_conduit to RPA_ between 4D Flow MRI and CFD showed r = 0.49, y = 0.38x + 41.50, bias = 8.73, and *p* < 0.001 respectively; and the Bland-Altman limits of agreement for PFD_conduit to RPA_ from the 4D Flow MRI compared to CFD was −8.7% ± 42.0% (Figure 7B).

**TABLE 3:** Results of pulmonary flow distribution for 4D Flow MRI and CFD.

Case	4D Flow MRI						CFD					
	SVC to LPA [%]	SVC to RPA [%]	Conduit to LPA [%]	Conduit to RPA [%]	Total to LPA [%]	Total to RPA [%]	SVC to LPA [%]	SVC to RPA [%]	Conduit to LPA [%]	Conduit to RPA [%]	Total to LPA [%]	Total to RPA [%]
Case 1	73.6	26.4	1.1	98.9	39.0	61.0	83.8	16.2	18.0	82.0	26.0	74.0
Case 2	1.1	98.9	62.8	37.2	51.7	48.3	25.0	75.0	60.7	39.3	23.6	76.4
Case 3	20.2	79.8	100.0	0.0	59.5	40.5	42.2	57.8	68.7	31.3	46.7	53.3
Case 4	88.7	11.3	36.2	63.8	43.3	56.7	74.1	25.9	30.6	69.4	61.0	39.0
Case 5	86.8	13.2	4.0	96.0	68.2	31.8	42.5	57.5	80.5	19.5	38.1	61.9
Case 6	67.6	32.4	0.0	100.0	38.6	61.4	74.0	26.0	17.6	82.4	58.3	41.7
Case 7	99.5	0.5	10.9	89.1	57.0	43.0	99.4	0.6	20.0	80.0	66.2	33.8
Case 8	78.4	21.6	6.0	94.0	57.7	42.3	63.2	36.8	53.8	46.2	40.5	59.5
Case 9	12.6	87.4	71.4	28.6	58.0	42.0	29.4	70.6	74.3	25.7	32.6	67.4
Case 10	34.6	65.4	56.8	43.2	29.7	70.3	46.2	53.8	18.3	81.7	39.3	60.7
Case 11	53.4	46.6	85.3	14.7	62.2	37.8	33.2	66.8	79.8	20.2	59.5	40.5
Case 12	3.1	96.9	72.9	27.1	38.0	62.0	16.4	83.6	51.8	48.2	35.6	64.4
Case 13	5.7	94.3	87.7	12.3	48.4	51.6	37.2	62.8	55.1	44.9	34.7	65.3
Case 14	0.4	99.6	80.9	19.1	38.8	61.2	33.9	66.1	40.4	59.6	47.2	52.8
Case 15	96.2	3.8	4.4	95.6	30.5	69.5	74.1	25.9	12.0	88.0	67.2	32.8
Case 16	16.1	83.9	18.0	82.0	14.0	86.0	0.0	100.0	20.5	79.5	17.4	82.6
Case 17	48.7	51.3	23.2	76.8	35.6	64.4	82.2	17.8	11.2	88.8	38.8	61.2
Case 18	5.4	94.6	100.0	0.0	62.1	37.9	26.7	73.3	77.9	22.1	20.0	80.0
Case 19	12.3	87.7	43.7	56.3	23.3	76.7	35.4	64.6	19.1	80.9	25.1	74.9
Case 20	60.5	39.5	51.3	48.7	21.5	78.5	42.5	57.5	4.5	95.5	37.6	62.4
Case 21	7.1	92.9	42.6	57.4	17.0	83.0	21.3	78.7	14.6	85.4	18.0	82.0
Case 22	62.5	37.5	44.9	55.1	31.8	68.2	78.7	21.3	7.4	92.6	51.7	48.3
Case 23	20.2	79.8	0.1	99.9	17.2	82.8	52.7	47.3	0.7	99.3	4.6	95.4
Case 24	4.0	96.0	95.9	4.1	49.6	50.4	18.5	81.5	69.3	30.7	43.0	57.0
Case 25	73.8	26.2	28.9	71.1	43.4	56.6	82.6	17.4	25.6	74.4	52.7	47.3
Case 26	24.6	75.4	100.0	0.0	25.8	74.2	38.8	61.2	18.0	82.0	27.5	72.5
Case 27	71.2	28.8	0.7	99.3	54.7	45.3	30.4	69.6	66.6	33.4	57.1	42.9
Case 28	6.6	93.4	100.0	0.0	56.2	43.8	6.5	93.5	89.0	11.0	64.6	35.4
Case 29	23.0	77.0	46.7	53.3	24.7	75.3	41.0	59.0	17.2	82.9	31.0	69.0
Mean ± SD	39.9 ± 33.6	60.1 ± 33.6	47.5 ± 36.2	52.5 ± 36.2	41.3 ± 15.7	58.7 ± 15.7	45.9 ± 25.7	54.1 ± 25.7	38.7 ± 27.8	61.3 ± 27.8	40.2 ± 16.4	59.8 ± 16.4

CFD, computational fluid dynamics; LPA, left pulmonary artery; RPA, right pulmonary artery; SVC, superior vena cava.

### 3.3 Regional parameters for 4D Flow MRI

The methods and results of regional parameters for 4D Flow MRI are summarized in Figures 2C, D and Table 4. The mean ± SD of V_mean_ were 0.15 ± 0.02 m/s at the SVC, 0.08 ± 0.01 m/s at the conduit, 0.17 ± 0.04 m/s at the LPA and 0.20 ± 0.06 m/s at the RPA, respectively. The KE was 0.04 ± 0.02 mJ at the SVC, 0.03 ± 0.01 mJ at the conduit, 0.03 ± 0.02 mJ at the LPA, and 0.03 ± 0.01 mJ at the RPA, respectively; and the VD was 0.03 ± 0.01 mW at the SVC, 0.03 ± 0.02 mW at the conduit, 0.03 ± 0.01 mW at the LPA and 0.02 ± 0.01 mW at the RPA, respectively. The diameter was 9.74 ± 1.43 mm at the SVC, 13.90 ± 1.51 mm at the conduit, 8.18 ± 1.55 mm at the LPA, and 8.15 ± 1.29 mm at the RPA, respectively.

**TABLE 4:** Results of hemodynamic parameters for 4D Flow MRI by region.

Case	V_mean_ [m/s]				KE [mJ]				VD [mW]				Diameter [mm]			
	SVC	Conduit	LPA	RPA	SVC	Conduit	LPA	RPA	SVC	Conduit	LPA	RPA	SVC	Conduit	LPA	RPA
Case 1	0.16	0.07	0.16	0.25	0.04	0.02	0.03	0.03	0.03	0.01	0.02	0.02	9.29	14.86	7.44	9.12
Case 2	0.19	0.09	0.14	0.19	0.07	0.03	0.04	0.04	0.04	0.02	0.02	0.03	10.14	14.70	10.49	9.64
Case 3	0.13	0.07	0.16	0.13	0.02	0.02	0.04	0.02	0.02	0.02	0.03	0.01	10.38	14.81	9.36	9.28
Case 4	0.15	0.07	0.21	0.19	0.03	0.02	0.05	0.03	0.02	0.02	0.05	0.03	10.33	15.24	9.80	9.06
Case 5	0.13	0.07	0.17	0.10	0.03	0.02	0.02	0.02	0.02	0.01	0.02	0.01	10.70	13.94	8.32	9.04
Case 6	0.15	0.07	0.16	0.17	0.03	0.02	0.02	0.02	0.02	0.02	0.02	0.02	7.54	14.69	9.45	7.80
Case 7	0.11	0.07	0.22	0.16	0.02	0.02	0.08	0.02	0.02	0.02	0.07	0.02	12.49	15.67	9.30	6.74
Case 8	0.18	0.06	0.21	0.20	0.08	0.01	0.03	0.02	0.05	0.02	0.02	0.02	9.60	12.35	9.92	7.05
Case 9	0.15	0.09	0.17	0.23	0.03	0.05	0.03	0.04	0.02	0.03	0.03	0.04	10.64	16.60	7.60	7.61
Case 10	0.16	0.07	0.13	0.19	0.03	0.02	0.02	0.02	0.02	0.04	0.03	0.02	9.87	14.11	8.56	8.73
Case 11	0.10	0.08	0.14	0.14	0.02	0.03	0.03	0.01	0.02	0.05	0.03	0.01	10.36	15.63	7.87	6.79
Case 12	0.15	0.08	0.10	0.23	0.05	0.02	0.01	0.03	0.03	0.02	0.01	0.03	9.83	14.54	10.55	8.54
Case 13	0.16	0.10	0.16	0.24	0.06	0.06	0.04	0.04	0.04	0.07	0.04	0.03	9.81	14.06	11.30	9.96
Case 14	0.15	0.08	0.23	0.27	0.03	0.03	0.03	0.03	0.02	0.05	0.03	0.04	8.42	15.17	6.54	7.09
Case 15	0.13	0.06	0.22	0.12	0.02	0.02	0.02	0.00	0.03	0.02	0.03	0.01	10.17	12.63	5.77	7.35
Case 16	0.17	0.09	0.12	0.26	0.04	0.03	0.01	0.05	0.02	0.03	0.01	0.05	6.95	12.71	6.52	10.94
Case 17	0.15	0.09	0.22	0.21	0.03	0.02	0.03	0.04	0.01	0.01	0.01	0.01	10.20	12.67	7.89	7.77
Case 18	0.17	0.09	0.15	0.21	0.06	0.03	0.03	0.02	0.04	0.04	0.03	0.02	10.42	11.82	6.38	7.79
Case 19	0.15	0.04	0.11	0.21	0.03	0.01	0.01	0.02	0.02	0.00	0.00	0.01	9.83	13.34	8.79	7.39
Case 20	0.14	0.07	0.20	0.24	0.04	0.02	0.01	0.03	0.04	0.02	0.01	0.04	6.29	12.73	6.71	6.14
Case 21	0.13	0.09	0.11	0.16	0.03	0.03	0.01	0.02	0.02	0.02	0.01	0.02	10.75	10.72	7.65	9.88
Case 22	0.16	0.09	0.24	0.31	0.06	0.06	0.05	0.04	0.05	0.04	0.05	0.03	11.68	16.36	7.93	6.46
Case 23	0.15	0.10	0.13	0.18	0.06	0.05	0.01	0.02	0.04	0.05	0.02	0.03	7.49	13.71	6.27	8.20
Case 24	0.16	0.09	0.14	0.26	0.06	0.04	0.03	0.02	0.03	0.05	0.02	0.02	9.00	13.76	9.94	8.36
Case 25	0.12	0.07	0.20	0.15	0.02	0.03	0.04	0.01	0.02	0.04	0.04	0.01	9.87	15.60	8.56	6.84
Case 26	0.16	0.08	0.12	0.23	0.04	0.02	0.01	0.02	0.02	0.03	0.01	0.01	9.14	11.74	5.91	6.51
Case 27	0.18	0.08	0.18	0.15	0.05	0.03	0.02	0.02	0.03	0.04	0.02	0.02	8.38	13.06	6.62	10.02
Case 28	0.20	0.10	0.12	0.20	0.11	0.04	0.02	0.03	0.07	0.08	0.03	0.02	12.02	11.54	6.63	9.35
Case 29	0.18	0.08	0.17	0.35	0.06	0.02	0.02	0.06	0.04	0.04	0.02	0.06	10.84	14.24	9.07	6.84
Mean ± SD	0.15 ± 0.02	0.08 ± 0.01	0.17 ± 0.04	0.20 ± 0.06	0.04 ± 0.02	0.03 ± 0.01	0.03 ± 0.02	0.03 ± 0.01	0.03 ± 0.01	0.03 ± 0.02	0.03 ± 0.01	0.02 ± 0.01	9.74 ± 1.43	13.90 ± 1.51	8.18 ± 1.55	8.15 ± 1.29

LPA, left pulmonary artery; KE, kinetic energy; RPA, right pulmonary artery; SVC, superior vena cava; V_mean_, mean velocity; VD, viscous dissipation.

## 4 Discussion

The purpose of this study was to assess baseline hemodynamics of Fontan circulation using 4D Flow MRI and CFD. Additionally, the correlation and discrepancies between 4D Flow MRI and CFD were analyzed. The main findings were as follows: 1) PFD from the SVC was strongly correlated. However, PFD from the conduit showed inferior correlation mostly due to a low velocity to noise ratio. 2) Velocity fields and KE were relatively similar. 3) VD from CFD were significantly larger than those from 4D Flow MRI in all cases.

### 4.1 Baseline hemodynamics in the patients with the Fontan procedure

In this study, PFD to LPA was 41.3% ± 15.7% according to 4D Flow MRI and 40.2 ± 16.4 according to CFD, respectively. Haggerty et al. (Haggerty et al., 2014) performed steady-state CFD simulations based on CMR data for 100 Fontan patients with ages ranging 12.0 ± 6.8 years, and PFD to LPA was 45% ± 12%. Additionally, Tang et al. (2014) performed CFD simulations using time-averaged boundary conditions based on CMR data for 108 Fontan patients with age ranging 10.2 ± 6.8 years, and PFD to LPA was 43% ± 12%; Frieberg et al. (2022) also performed transient and steady-state CFD simulations based on MRI data for nine Fontan patients with age ranging 9.2 ± 5.6 years, and PFD to LPA was 38% ± 11% according to MRI, 37% ± 9.5% according to pulsatile CFD, and 37% ± 9.5% according to steady-state CFD, respectively. Although the values differ among these studies, they were similar overall. As the number of congenital heart disease patients has increased in recent decades, studies on complex anatomical vascular structures and the resulting hemodynamic changes have been increasing, highlighting the importance of co-validate with 4D Flow MRI and CFD (Sjöberg et al., 2017). In this study, KE was 0.15 ± 0.04 mJ in 4D Flow MRI and 0.12 ± 0.05 mJ in CFD, and there was no significant difference between 4D Flow MRI and CFD. However, the VD was 0.14 ± 0.04 mW according to 4D Flow MRI and 0.59 ± 0.30 mW according to CFD in this study. In their previous study, Cibis et al. (2015) performed transient CFD based on 4D Flow MRI for six Fontan patients aged between 9 and 21, and based on CFD results using a 0.6 mm mesh size, spatial resolution was mapped to voxels of the equal size, down-sampled to calculate VD, and then compared with values from 4D Flow MRI. VD was 0.81 ± 0.55 mW according to CFD, 0.49 ± 0.26 mW according to down-sampling, and 0.56 ± 0.28 mW according to 4D Flow MRI, respectively. It was confirmed that as the spatial resolution decreased, the VD also decreased, and the value of VD in CFD was higher than 4D Flow MRI. The regional diameters were 9.74 ± 1.43 at SVC, 13.90 ± 1.51 at the conduit, 8.18 ± 1.55 at the LPA, and 8.15 ± 1.29 at the RPA, respectively. In another study, Ha et al. (2019) reported on the diameters of 12 Fontan patients with age ranging 3.0 ± 0.4, and diameter was 8.1 ± 1.5 at SVC, 10.7 ± 2.1 at conduit, 7.1 ± 2.1 at LPA, and 7.4 ± 1.5 at RPA, respectively. Similar diameters appeared in similar age groups, and differences in values are to be expected from patient to patient; however, in our current study, the diameter was relatively larger because the diameter was obtained with 4D Flow MRI without smoothing.

### 4.2 Energetics in 4D Flow MRI and CFD

Quantification of PFD is crucial in the treatment of Fontan circulation since hepatic flow distribution has been found to be associated with the development of pulmonary arteriovenous malformations (Yang et al., 2012; Bächler et al., 2013). The quantification of PFD can be accomplished using two methods: the flow rate calculation method (Tang et al., 2014; Frieberg et al., 2022) and the calculation method using particle trace (Bächler et al., 2013; Ha et al., 2019; Weiss et al., 2023). We found a strong correlation (r = 0.71) between 4D Flow MRI and CFD for PFD from the SVC, while a moderate correlation (r = 0.40) was observed for PFD from the conduit. This difference can be attributed to the use of a conduit larger than the existing blood vessel size during the Fontan procedure, which leads to lower velocity due to its larger size, resulting in a lower V_mean_ compared to other regions (Table 4). As a consequence, it becomes more challenging to obtain accurate flow trajectories due to the lower velocity-to-noise ratio within the conduit. Moreover, the increased risk of thrombosis is associated with sluggish flow, rendering the implantation of larger conduit sizes in young children unfeasible and undesirable (Itatani et al., 2009). Instead, considering conduits made from alternative materials is also suggested as a viable approach, rather than relying solely on the use of larger conduits (Rijnberg et al., 2022a).

The missing fraction of streamline tracers using 4D Flow MRI was proposed as a useful index to evaluate the accuracy of flow distribution analysis (Ha et al., 2019). It should be noted that due to limited spatial resolution and the presence of velocity noise, a certain fraction of streamline tracers may cross the boundary of the vessel or abruptly stop at internal points. The percentage of missing particles when calculating PFD using 4D Flow MRI was 82.4% ± 10.0% in this study. The use of CFD based on 4D Flow MRI data can partially address the impact of limited spatial resolution and velocity noise on streamline tracing. This approach has the potential to enhance the clinical relevance of our findings.

In Fontan circulation, blood is typically circulated to the lungs passively without the assistance of a ventricle. Therefore, monitoring the kinetic energy (KE) related to blood flow energy is an important parameter (Kamphuis et al., 2021; Rijnberg et al., 2022b; Weiss et al., 2023). In this study, a moderate correlation (r = 0.60) was shown between the KE linear regression of 4D Flow MRI and CFD, and the velocity field showed similar results overall (Figure 3). In CFD, the KE was similar to or higher than that in 4D Flow MRI, and the average KE was 20% higher than CFD (Table 2). Changes in V_max_ in both caval veins were found to have a strong correlation with changes in total energy loss and mean energy loss rate (Weiss et al., 2023). Therefore, accurate measurement of V_max_ is essential for evaluating energy loss in Fontan circulation. However, the mean V_max_ according to 4D Flow MRI was approximately 1.5 times higher than CFD in this study (Table 2). This discrepancy could be attributed to differences in spatial resolution and inaccurate boundary conditions between the two methods. Nevertheless, there was no significant difference between KE and V_max_, as CFD was based on time-averaged 4D Flow MRI data, and the noise problem of the conduit was reasonably good for the assumption that the boundary condition of the conduit was a pressed-inlet for mass conservation in CFD.

VD has been associated with clinical outcomes in Fontan patients (Kamphuis et al., 2019; Kamphuis et al., 2021; Rijnberg et al., 2022a), making it a hemodynamic metric of clinical interest. The VD linear regressions according to 4D Flow MRI and CFD both showed a moderate correlation (r = 0.40) and were the lowest among all parameters compared in this study. In all cases, this value according to CFD was higher than that of 4D Flow MRI, and there was an average difference of four times. The reason for this was that the equation for VD is related to the gradient of space (Cibis et al., 2015), where the spatial resolution is 2 mm for 4D Flow MRI and 0.4 mm for CFD. Accurate comparison of 4D Flow MRI and CFD is essential for determining the clinical applicability of VD in Fontan patients. However, achieving an accurate comparison is challenging due to differences in spatial resolution between the two methods.

The issue of segmentation in the Fontan circulation warrants attention. The question of whether segmentation was performed in a standardized manner, including the level of segmental branches, remains a valid concern. Normalization of energetics was employed to facilitate meaningful comparisons between different segments based on centerlines and confluence (Rijnberg et al., 2021). In this study, segmentation was performed based on the phase-contrast and magnitude images obtained from 4D Flow MRI. The segmentation process was validated through intra- and inter-observer assessments. By utilizing the spatial mean values, the study aimed to evaluate the overall flow and energetics of the Fontan circulation and provide valuable insights into its fluid dynamics. This approach allowed for a comprehensive assessment of the circulation and facilitated the characterization of its fluid properties. Normalization of energetics is worth considering the inclusion of such normalization techniques in future studies to enhance the comparability of results and provide a more comprehensive understanding of the energetics within the Fontan circulation.

### 4.3 Limitations

This study has several limitations. First, the fluid was assumed to be Newtonian. While many studies on Fontan circulation have considered non-Newtonian fluid models for CFD analysis (Wei et al., 2020; Frieberg et al., 2022; Prather et al., 2022), the Newtonian model was used in this study to ensure consistency in calculating viscous dissipation between 4D Flow MRI and CFD. Second, the vessel wall was assumed to be rigid. However, this assumption is an essential characteristic of CFD only, and is used in all CFD studies on Fontan circulation. Third, atmospheric pressure was applied as the pressure-inlet to the boundary condition of the conduit. Due to the presence of noise effects, the total flow rates in the SVC, LPA, RPA, and conduit did not adhere to mass conservation. To address this, mass flow was employed for SVC, LPA, and RPA, while pressure conditions were utilized in cases with high noise levels to ensure continuity. As the analysis focused solely on the flow field under rigid body vessel conditions, the pressure0inlet does not exert any influence on the results. Fourth, a wide Venc range of 40–150 cm/s was used for 4D Flow MRI in this study. While a lower Venc range of 40–80 cm/s was used for most cases, a relatively high value of 150 cm/s was used in only one case where aliasing occurred. However, a high Venc value may result in underestimation of the peak velocities and may affect the accuracy of the flow measurements. Therefore, it is important to carefully select the appropriate Venc range based on the expected flow velocities for each case.

Lastly, our study is that the use of ECG-gated data in 4D Flow MRI completely ignores the effect of respiration on flow. This may result in the missed observation of retrograde flow in some patients, particularly in very young Fontan patients with oversized conduits. Although our study used 4D Flow MRI to assess the flow patterns in Fontan patients, the lack of consideration of respiratory-induced flow changes could have led to underestimation of retrograde flow in some cases. Future studies may consider incorporating respiratory gating techniques or other methods to better account for the effect of respiration on flow.

## 5 Conclusion

4D Flow MRI was performed and served as the basis for steady-state CFD immediately following the Fontan procedure, and the resulting data were compared with data from 4D Flow MRI. PFD showed a strong linear correlation in the SVC, but a relatively weak correlation in conduit due to noise problems was caused by low velocity. The overall velocity fields and KE were similar between the two imaging modalities, but due to the difference in spatial resolution in VD, CFD showed a larger value than 4D Flow MRI for all cases. This study established a baseline for patients immediately following the Fontan procedure, and confirmed correlation and discrepancy via comparison with CFD. We plan to conduct a follow-up study in the future based on the baseline data from this study to observe characteristics and changes.

## Data Availability

The original contributions presented in the study are included in the article/Supplementary Material, further inquiries can be directed to the corresponding authors.

## Abbreviations

4D Flow MRI, four-dimensional flow magnetic resonance imaging; CFD, computational fluid dynamics; CMR, cardiac magnetic resonance; KE, kinetic energy; LPA, left pulmonary artery; PFD, pulmonary flow distribution; RPA, right pulmonary artery; SD, standard deviation; SVC, superior vena cava; VD, viscous dissipation.
