# Supplementary material for: Characterization of baseline hemodynamics after the Fontan procedure: a retrospective cohort study on the comparison of 4D Flow MRI and computational fluid dynamics
Source: Front Physiol. 2023 May 25;14:1199771. doi: 10.3389/fphys.2023.1199771 (PMC10248477; doi:10.3389/fphys.2023.1199771)
Supplement: Supplementary file 1 [file DataSheet1.docx]

**Characterization of baseline hemodynamics after the Fontan procedure: A retrospective cohort study on the comparison of 4D Flow MRI and computational fluid dynamics**

Gyu-Han Lee^1^ · Hyun Jung Koo^3^ · Kyung Jin Park^3,4^ · Dong Hyun Yang^3†^ · Hojin Ha^2†^

^1^ Department of Interdisciplinary Program in Biohealth-Machinery Convergence Engineering, Kangwon National University, 1, Kangwondaehak-gil, Chuncheon 24341, Republic of Korea

^2^ Department of Smart Health Science and Technology, Kangwon National University, 1, Kangwondaehak-gil, Chuncheon 24341, Republic of Korea

^3^ Department of Radiology and Research Institute of Radiology, Asan Medical Center, University of Ulsan College of Medicine, 88, Olympic-ro 43-gil, Songpa-gu, Seoul 05505, Republic of Korea

^4^ Department of Electrical and Electronic Engineering, Yonsei University, 50, Yonsei-ro, Seodaemun-gu, Seoul 03722, Republic of Korea

^†^ These authors contributed equally to this work.

**Corresponding author**

Dong Hyun Yang

Department of Radiology and Research Institute of Radiology, Asan Medical Center, University of Ulsan College of Medicine, 88, Olympic-ro 43-gil, Songpa-gu, Seoul 05505, Republic of Korea. Tel: +82-2-3010-5820; e-mail: donghyun.yang@gmail.com

Hojin Ha

Department of Smart Health Science and Technology, Kangwon National University, 1 Kangwondaehak-gil, Chuncheon 24341, Republic of Korea. Tel: +82-33-250-6310; e-mail: hojinha@kangwon.ac.kr

**Word count:** 3331

**Number of figures and tables:** 7 figures and 4 tables

**Fig. S1** Mesh dependency test


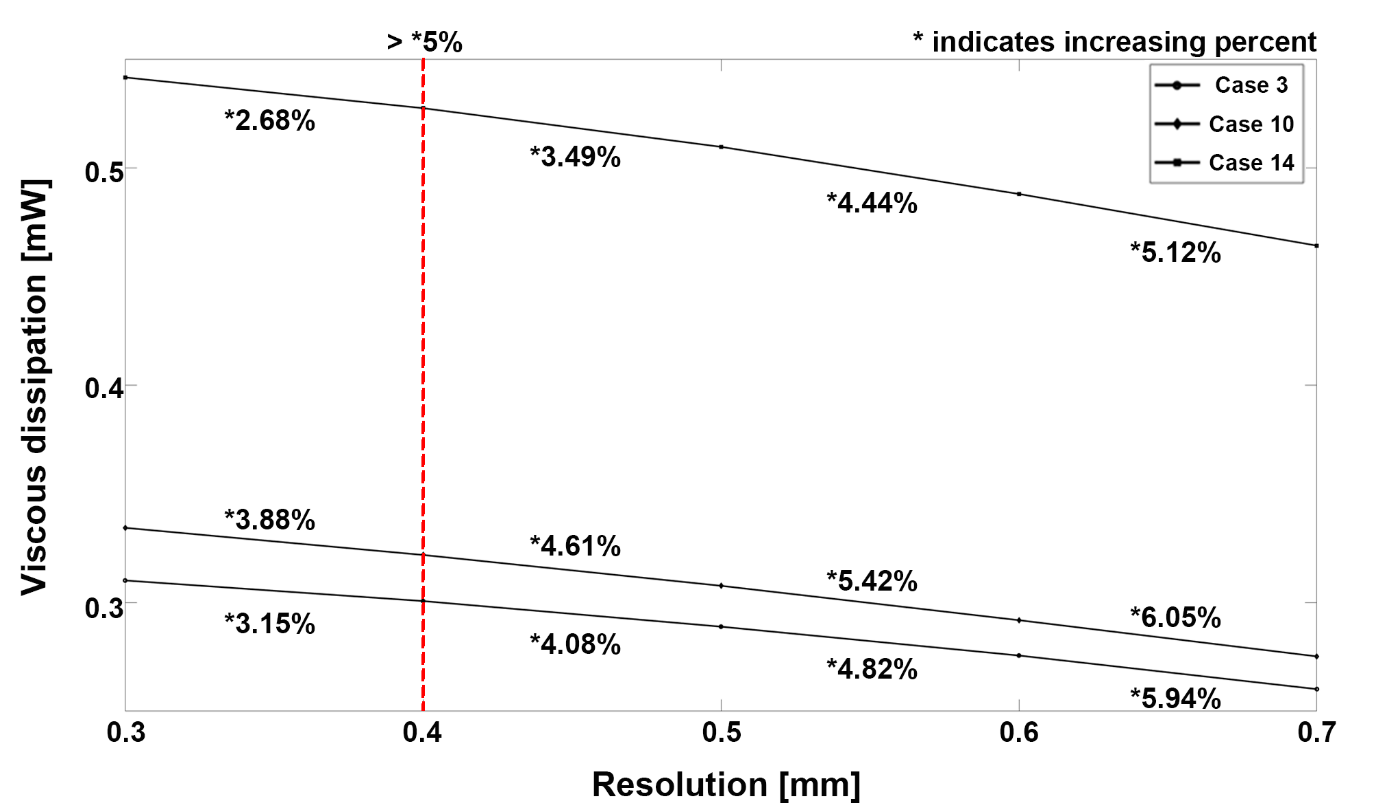


**Fig. S2** Correlation plot and Bland-Altman plot for intra-observer variability.

**a.** Kinetic energy. **b.** Viscous dissipation


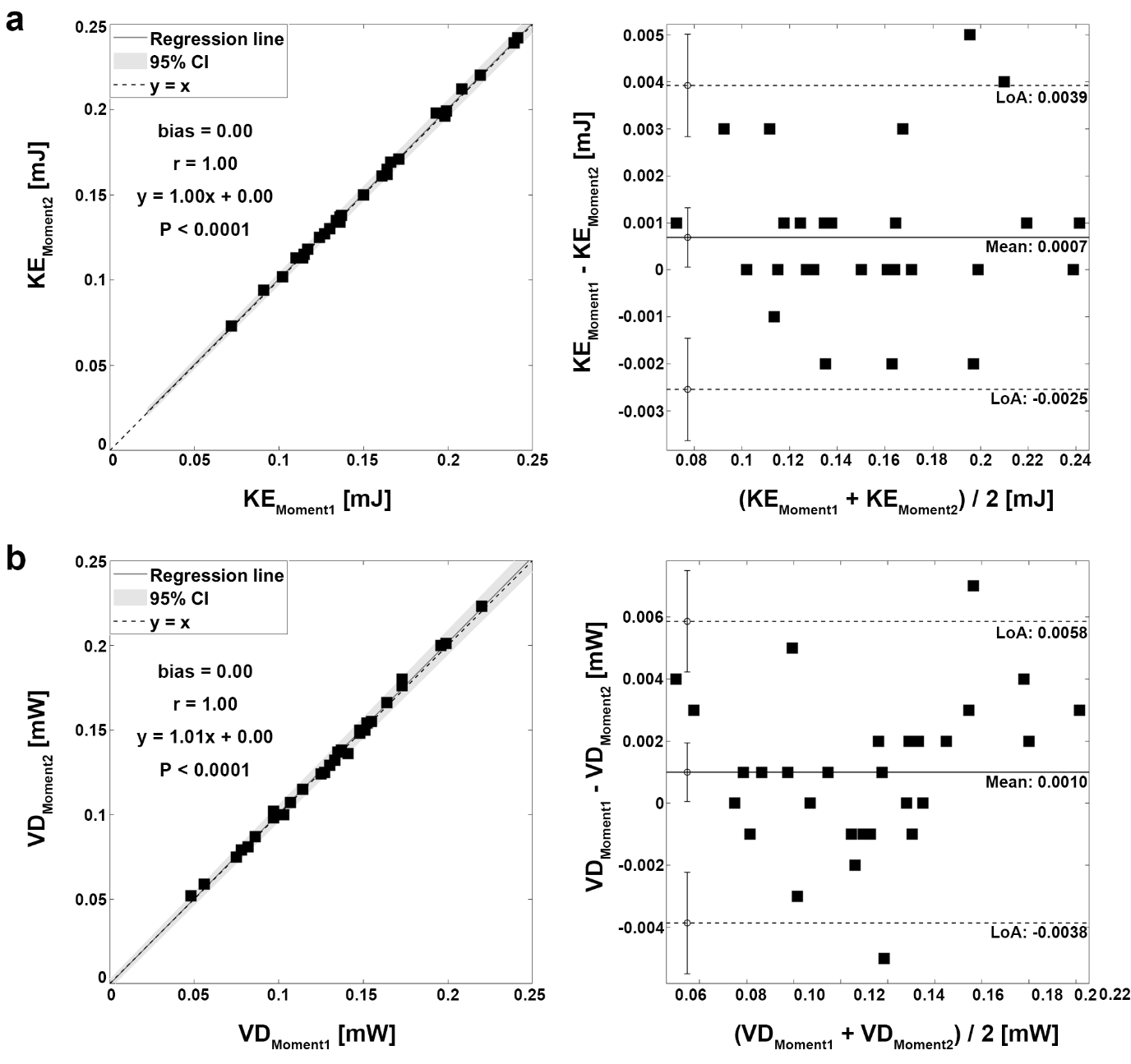


Fig. S2 showed the result of intra-observer reproducibility. The linear regression analysis of KE between two intra-observer moments showed r=1.00, y=1.00x + 0.00, bias=0.00, and p<0.0001, respectively. The Bland-Altman limits of agreement of KE from the two intra-observer moments was 0.0007 ± 0.0032 mJ. The linear regression analysis of VD between two intra-observer moments in showed r=1.00, y=1.01x + 0.00, bias=0.00, and p<0.0001, respectively. The Bland-Altman limits of agreement of VD from the two intra-observer moments was 0.0010 ± 0.0048 mW.

**Fig. S3** Correlation plot and Bland-Altman plot for inter-observer variability.

**a.** Kinetic energy. **b.** Viscous dissipation


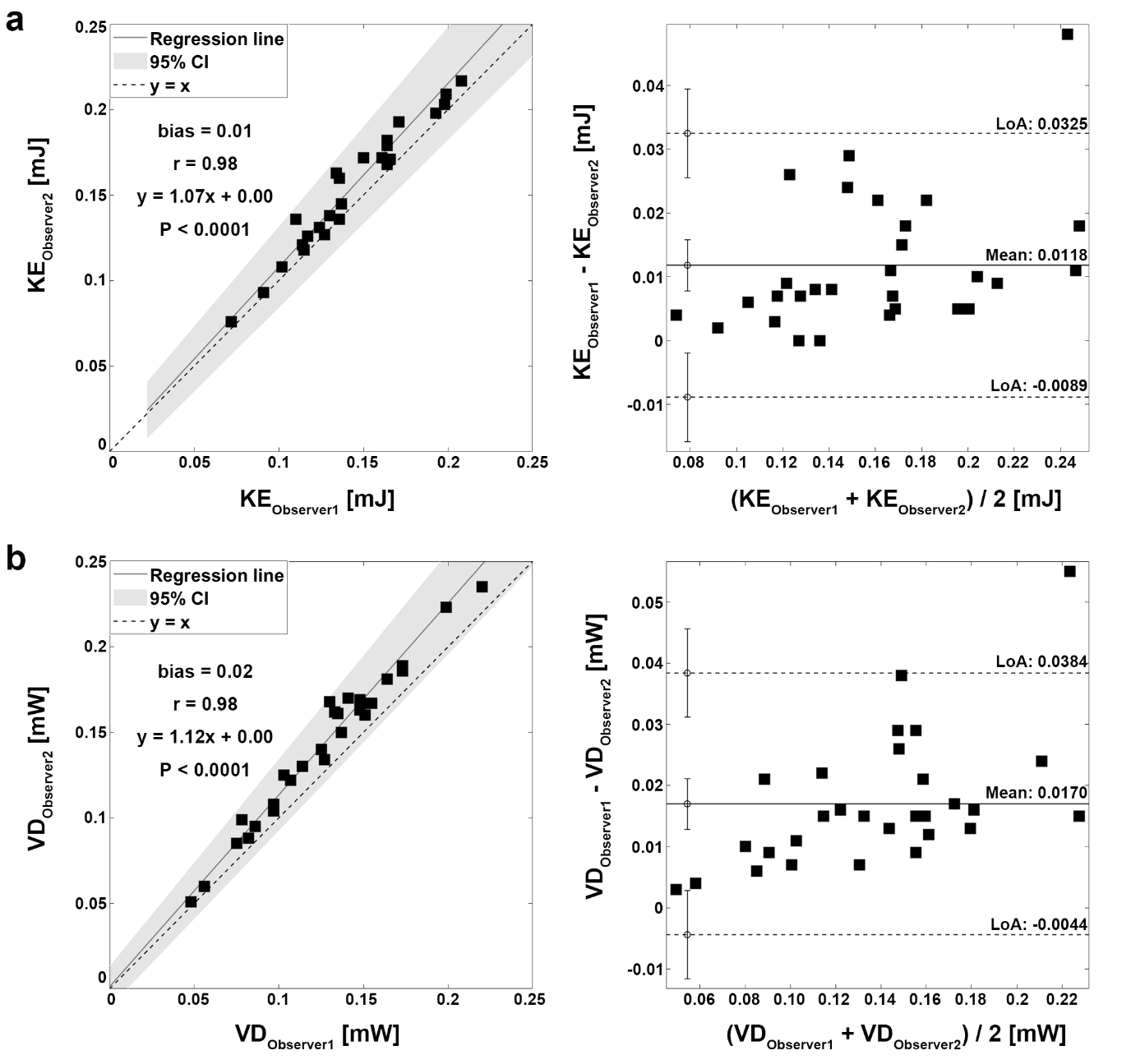


Fig. S3 showed the result of inter-observer reproducibility. The linear regression analysis of KE between two different observers showed r=0.98, y=1.07x + 0.00, bias=0.01, and p<0.0001, respectively. The Bland-Altman limits of agreement of KE from the two different observers was 0.0118 ± 0.0207 mJ. The linear regression analysis of VD between two intra-observer moments in showed r=0.98, y=1.12x + 0.00, bias=0.02, and p<0.0001, respectively. The Bland-Altman limits of agreement of VD from the two intra-observer moments was 0.0170 ± 0.0214 mW.
